# Complete hydatidiform mole in one twin after embryo transfer: a case report and literature review

**DOI:** 10.3389/fmed.2026.1934255

**Published:** 2026-09-04

**Authors:** Zeru Hou, Yanan Zhao, Shutong Wei, Jiarong Wang, Jianjun Zhang, Yan Wang

**Affiliations:** 1 The Affiliated Hospital of Shandong Second Medical University, Shandong Second Medical University, Weifang, China; 2 The Affiliated Hospital of Shandong Second Medical University, Weifang Key Laboratory of Postpartum Pelvic Floor Electromyographic Rehabilitation, Weifang Key Laboratory of Precision Diagnosis and Treatment of Adverse Pregnancy, Weifang, China

**Keywords:** complete hydatidiform mole in one twin, gestational trophoblastic disease, *in vitro* fertilization–embryo transfer, pregnancy outcomes, twin gestation

## Abstract

A complete hydatidiform mole coexisting with a normal fetus in a twin gestation (CHMCF) is an extremely rare form of abnormal multifetal gestation characterized by the coexistence of a normal fetus and its placenta alongside a complete hydatidiform mole, with a markedly increased risk of persistent gestational trophoblastic disease (PTD) in the mother. Embryo transfer, as a key procedure in assisted reproductive technology, achieves conception by transferring *in vitro*-fertilized embryos into the uterine cavity. Although it addresses infertility, it may also present distinct clinical scenarios involving rare gestational complications. This report describes a case of a complete hydatidiform mole coexisting with a fetus in a twin gestation after embryo transfer. By integrating serial changes in serum β-human chorionic gonadotropin (β-hCG) levels, ultrasonographic features, chromosomal analysis, and histopathological findings, we summarize the diagnostic and management course and review the relevant literature.

## Introduction

A complete hydatidiform mole (CHM) is an abnormal gestation characterized by aberrant proliferation of trophoblastic cells and edema of the villous stroma, typically without fetal tissue (1). Its incidence varies by region, ranging from approximately 0.5 to 2 per 1,000 gestations (2, 3). The condition in which one fetus in a twin gestation is a complete hydatidiform mole and the other is a normal fetus (twin gestation with complete hydatidiform mole and coexisting normal fetus, CHMCF) is even rarer, with an incidence of approximately 1 in 20,000 to 100,000 gestations (4, 5). Its clinical course is complex, and clinical management is challenging.

In recent years, with the increasing use of assisted reproductive technology (ART), the incidence of twin gestations has increased; however, cases of CHMCF after ART remain uncommon (6). Such gestations carry multiple risks, including persistent gestational trophoblastic disease (PTD), preeclampsia, hyperthyroidism, bleeding, and preterm birth, while also requiring careful monitoring of the growth and safety of the normal fetus (7, 8). Therefore, early diagnosis, intensive surveillance, and multidisciplinary management are essential for optimizing maternal and fetal outcomes.

This case report describes a case of a complete hydatidiform mole coexisting with a fetus in a twin gestation after *in vitro* fertilization and embryo transfer (IVF-ET). This report aims to examine the clinical characteristics, key imaging and pathological diagnostic features, management strategies, and gestational outcomes, to provide practical guidance for the clinical recognition and management of this rare entity.

## Case report

**Figure 1:**
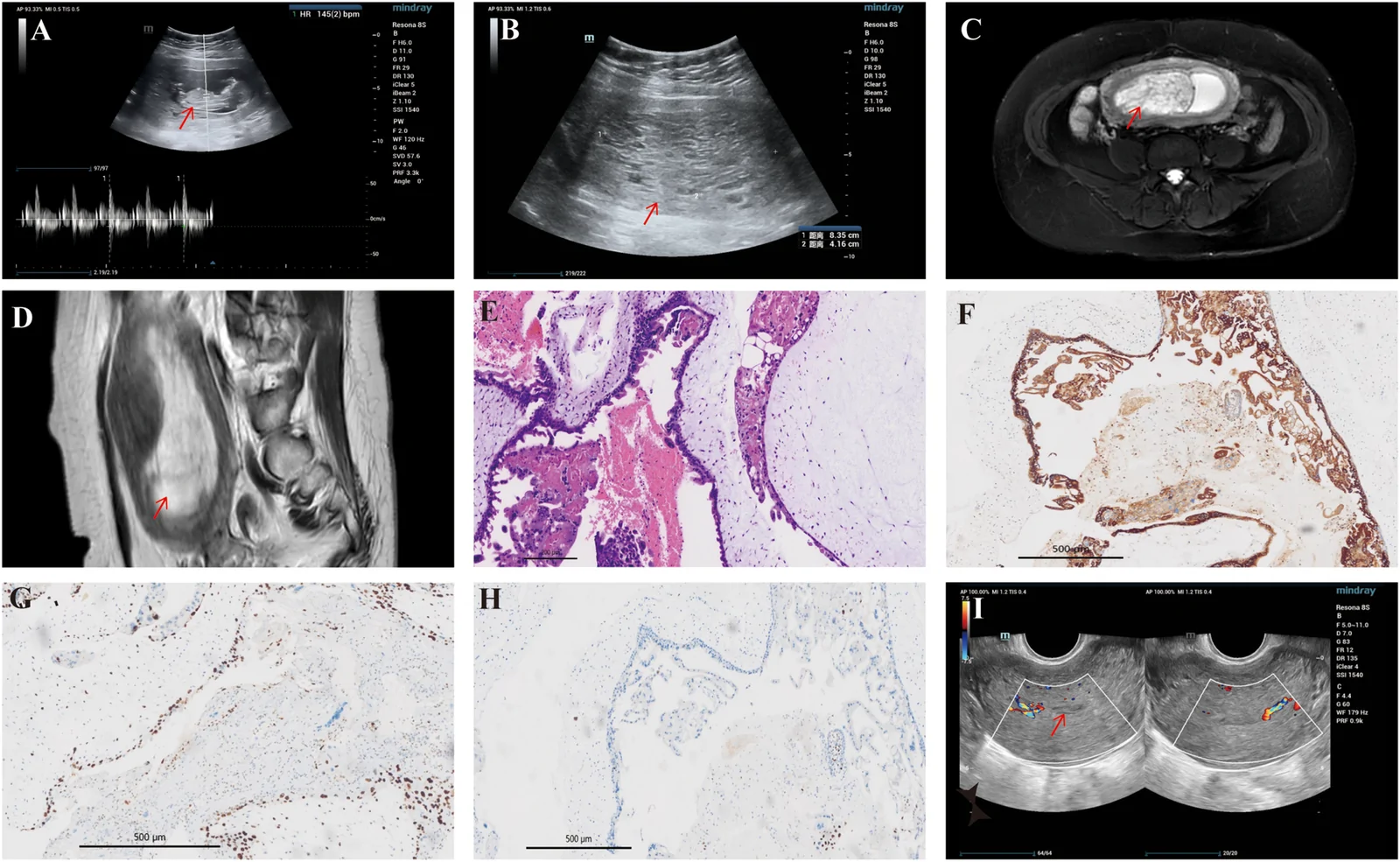
Imaging and pathological findings of the patient. **(A)** Preoperative ultrasound on August 16, 2025 shows a viable fetus within the uterine cavity, with identifiable cardiac activity and limb structures, confirming intrauterine pregnancy. The red arrow indicates the normal fetus. **(B)** Preoperative ultrasound on the same date reveals a typical honeycomb-like anechoic area in the placental region, exhibiting a “snowstorm” sign, consistent with the sonographic features of complete hydatidiform mole. The red arrow indicates the molar tissue (honeycomb-like anechoic area). **(C)** Axial pelvic MRI shows a mixed-signal lesion in the right side of the uterine cavity. The red arrows indicate the mixed-signal lesion in the right uterine cavity (molar tissue). **(D)** Sagittal pelvic MRI further delineates the intrauterine pregnancy and the abnormal signal in the right cavity, consistent with the axial view. The red arrows indicate the mixed-signal lesion in the right uterine cavity (molar tissue). **(E)** Hematoxylin and eosin (H&E) staining shows edematous villi and marked trophoblastic proliferation, suggesting histological features of hydatidiform mole. **(F)** Immunohistochemical staining for inhibin reveals diffuse positive expression in trophoblastic cells (scale b*a*r = 500 μm). **(G)** Immunohistochemical staining for Ki-67 shows a markedly elevated proliferation index, reflecting active tumor cell proliferation (scale bar = 500 μm). **(H)** Immunohistochemical staining for p57 demonstrates negative expression, providing molecular evidence for the diagnosis of complete hydatidiform mole (scale bar = 500 μm). **(I)** Ultrasound performed 10 days postoperatively (September 1, 2025) demonstrates a heterogeneous hyperechoic area within the uterine cavity, with punctate and linear blood flow signals. The red arrow indicates the heterogeneous hyperechoic area within the uterine cavity.

A 37-year-old multiparous patient was admitted to our hospital at 13 weeks and 5 days' gestation after ultrasonography suggested a hydatidiform mole in one of her twins. She had a history of regular menstrual cycles since age 14 with cycle intervals of 28–30 days. Her last menstrual period was May 15, 2025.The patient had no significant past medical history. She underwent a medical abortion for an unplanned pregnancy in 2010 and had a term vaginal delivery of a healthy female infant in 2012, and she remains healthy. In July 2024, hysterosalpingography at our hospital revealed bilateral tubal incomplete obstruction. In the same year, semen analysis of her male partner showed normal volume and liquefaction, low sperm concentration, normal sperm motility, and normal morphology. Chromosomal karyotyping of both partners revealed no abnormalities. In December 2024, the patient underwent *in vitro* fertilization (IVF) at our hospital due to secondary infertility, bilateral tubal incomplete obstruction, and moderate oligozoospermia in the male partner, using a gonadotropin-releasing hormone (GnRH) antagonist protocol. Nine oocytes were retrieved, and two day-3 embryos were cryopreserved. In June 2025, the two frozen-thawed day-3 embryos were transferred. Preimplantation genetic testing for aneuploidy (PGT-A) was not performed because there were no clinical indications for its use, and the embryos had been cryopreserved at the cleavage stage (day 3), which precluded trophectoderm biopsy. Serum β-human chorionic gonadotropin (β-hCG) testing 13 days after transfer confirmed conception. At 9 weeks and 2 days of amenorrhea, ultrasonography showed a single viable intrauterine fetus, with an anechoic fluid collection measuring approximately 2.2×2.3×0.7 cm surrounding the gestational sac, consistent with intrauterine fluid collection. At 13 + 2 weeks of gestation, ultrasonography showed a honeycomb-like heterogeneous echogenic area measuring approximately 8.4×6.3×4.2 cm on the right side of the uterine cavity, with punctate and linear vascular signals detected at the interface of the posterior uterine wall and myometrium (Figures 1A,B). Follow-up ultrasonography at another hospital showed a single viable intrauterine fetus, with a heterogeneous hypoechoic mass near the right uterine wall measuring approximately 10.4×5.9×5.2 cm, containing numerous densely distributed small cystic echoes in a honeycomb pattern. Peripheral vascular signals were partially detected, while only scattered stellate vascular signals were evident within the mass. Serum β-hCG exceeded 200,000 mIU/mL. A complete hydatidiform mole was suspected. After admission, a chest computed tomography (CT) scan showed a small pulmonary nodule in the right upper lobe. A pelvic magnetic resonance imaging (MRI) showed findings compatible with an intrauterine gestation (singleton); mixed-signal intensity was observed on the right side of the uterine cavity, and the possibility of a hydatidiform mole coexisting with a fetus could not be excluded. Multiple enlarged lymph nodes were noted in the pelvis and bilateral inguinal regions (Figures 1C,D). Thyroid stimulating hormone (TSH) was 0.01 μIU/mL, free thyroxine (free T4) was 24.9 pmol/L, and free triiodothyronine (free T3) was 7.54 pmol/L. Review of the patient's pregestational thyroid function showed no abnormalities, leading to a diagnosis of gestational transient thyrotoxicosis. No other clinically significant abnormalities were identified. A multidisciplinary case conference was subsequently held, and the consensus was that the patient's working diagnosis of a twin gestation (one viable fetus and one hydatidiform mole) was established. The patient subsequently underwent ultrasound-guided forceps curettage with uterine evacuation. Before the procedure, a water balloon was placed vaginally for cervical preparation. Hydrocortisone 100 mg, 5% glucose solution 500 mL, and vitamin C 0.5 g were administered intravenously to prevent thyroid storm, and cefuroxime 1.5 g was administered intravenously every 12 h for infection prophylaxis. During surgery, the uterine cavity depth was approximately 11 cm. Inspection showed that the fetal tissue and placenta were largely complete, along with vesicular tissue of variable sizes, which were submitted for histopathological examination. On postoperative day 1, β-hCG was 93,763 mIU/mL; on postoperative day 3, serum β-hCG was 15,559 mIU/mL. Histological examination of the hydatidiform villi showed edematous villi with trophoblastic hyperplasia and no fetal parts. Immunohistochemistry (EnVision two-step system, a commercial detection system) was performed with ethylenediaminetetraacetic acid (EDTA)-based heat-induced antigen retrieval and primary antibodies (all ready-to-use, Roche Diagnostics): anti-p57 (CDKN1C, clone KP10), anti-inhibin-α (clone EP378), and anti-Ki-67 (proliferation marker protein, clone MIB-1), followed by 3,3′-diaminobenzidine (DAB) chromogen and hematoxylin counterstaining, with appropriate positive and negative controls. Inhibin-α was positive in syncytiotrophoblasts; the Ki-67 proliferation index was approximately 70%; and p57 was negative in villous stromal and cytotrophoblast nuclei. These findings confirmed the diagnosis of complete hydatidiform mole. (Figures 1E–H). DNA from both fetal tissues was analyzed by copy number variation sequencing (CNV-seq) at 1-Mb resolution plus short tandem repeat (STR) for molar villi (performed by BGI-Shenzhen, Shenzhen Huada Medical Laboratory), and at 100-kb for normal villi. Reads were aligned to GRCh37/hg19 and interpreted according to American College of Medical Genetics and Genomics (ACMG) 2019 and Clinical Genome Resource (ClinGen) criteria. Molar villi exhibited diploid genome-wide uniparental disomy (UPD), indicating homozygosity (single allele peak) at all informative loci, consistent with androgenetic origin, without triploidy or pathogenic copy number variations (CNVs). Normal villi had three variants of uncertain significance (VUS): a 167.94 kb deletion at 5q23.1, a 168.14 kb duplication at 12p13.33, and a 138.63 kb duplication at 19q13.42; none of these were considered relevant to the fetal phenotype. Follow-up gynecological color Doppler ultrasonography performed on the 12th postoperative day showed a heterogeneous hyperechoic lesion measuring approximately 2.0×1.8×0.8 cm in the left side of the uterine cavity, with ill-defined margins. Color Doppler Flow Imaging (CDFI): Within the lesion, punctate and linear vascular signals measuring 1.2×1.2×0.6 cm were noted; peak systolic velocity (PSV): 38.7 cm/s (Figure 1I); The juxta-endometrial myometrium on the right wall showed heterogeneous echotexture; CDFI showed punctate and linear vascular signals measuring 1.0×2.2×0.7 cm, with a PSV of 25.9 cm/s. Serum β-hCG was 622 mIU/mL.Because of concern for incomplete evacuation of a hydatidiform mole, the patient was readmitted to undergo an ultrasound-guided evacuation combined with hysteroscopy. During the procedure, residual gestational tissue measuring approximately 2.0×2.0×1.0 cm was identified on the anterior wall of the uterine cavity. Under ultrasonography guidance, suction evacuation was carried out using an 8-gauge cannula, and the entire uterine cavity was curetted. On repeat inspection with the hysteroscope, no obvious residual tissue was noted, and the specimen was submitted for pathological testing. Postoperative pathology showed: Microscopic examination of the submitted specimen (endometrial contents) revealed proliferative endometrium, smooth muscle tissue, blood clots, and necrotic tissue, with some degenerated trophoblastic cells exhibiting enlarged, deeply stained nuclei. On the day after the second curettage (September 2), the serum β-hCG level decreased to 392 mIU/mL, and subsequently showed a consistent downward trend: 115 mIU/mL on September 9, 34.7 mIU/mL on September 16, and 14.2 mIU/mL on September 24. Subsequent telephone follow-up was performed to remind the patient of regular serum β-hCG testing, but the patient declined and further monitoring until serological normalization could not be completed. The diagnosis of CHM was based primarily on histopathology and immunohistochemistry; without parental DNA, STR genotyping provided supportive rather than conclusive evidence of androgenetic origin.

## Discussion

Histopathologically, CHM is defined by diffuse edema of the placental villi, variable degrees of trophoblastic hyperplasia, and the absence of embryonic or fetal tissue (9). Its genetic constitution is that of an androgenetic diploid conceptus, arising after fertilization of an “empty oocyte” (lacking maternal nuclear genetic material) by one sperm that duplicates its genome or by two sperm, resulting in complete absence of maternal genomic contribution (10, 11). Typical clinical manifestations include post-amenorrhea vaginal bleeding (most often occurring between 8 and 12 weeks of gestation), uterine enlargement greater than expected for gestational age, and severe hyperemesis gravidarum. Some patients develop gestational transient thyrotoxicosis because markedly elevated levels of β-hCG stimulate the thyroid gland (12). The diagnosis of CHM has evolved from traditional morphological identification toward a precision diagnostic model based on multimodal assessment, with a diagnostic framework integrating four dimensions: clinical presentation, imaging features, serological markers, and molecular pathology. Ultrasonography remains the primary screening modality, with typical findings including a “snowflake sign” or “honeycomb” pattern of mixed echogenicity within the uterine cavity, in the absence of fetal structures or an amniotic cavity (13). Serum β-hCG concentrations are often markedly elevated, frequently exceeding 100,000 mIU/mL (14, 15). Histopathological diagnosis remains the gold standard; on gross examination, vesicular tissue of variable sizes is evident, whereas microscopic features include villous edema, varying degrees of trophoblastic hyperplasia, and the absence of embryonic or fetal tissue. Immunohistochemical assessment of p57 is of major diagnostic value: CHM is typically p57-negative due to the absence of maternal genomic material, whereas partial hydatidiform mole (PHM) retains p57 positivity (9, 11, 16). In a 2026 study, Zhu et al. emphasized the importance of molecular genetic analysis in the precision diagnosis and management of hydatidiform moles, noting that it can accurately distinguish CHM (complete haplo-paternal origin) and PHM (poly-haplo-trisomy), and is currently a recommended method for precision diagnosis (17).

CHMCF is an exceptionally rare subtype of gestational trophoblastic disease, with a reported incidence of approximately 1 in 20,000 to 1 in 100,000 gestations (18). As shown in Table 1, Jones et al. first reported an incidence of 1 in 22,000 in a retrospective study of 8 cases in 1975 (19). This classic estimate has been widely cited. A recent systematic review published by Salmeri et al. in 2025 (incorporating 19 studies with a total of 417 CHMCF cases) confirmed this incidence range (5), representing the largest evidence base to date (Table 1).

**Table 1:** Summary Analysis of the Epidemiological Characteristics of CHMCF.

Research	Year	Number of cases	Key epidemiological findings
Jones et al. (19)	1975	8	Incidence rate: 1 in 22,000
Matsui et al. (Japan National Collaborative Study) (18)	2000	72	The incidence of persistent trophoblastic disease following a genetically confirmed complete molar pregnancy with a viable fetus is 50%.
Sharon Zilberman et al. (20)	2019	3 (Literature review: 248 cases)	Among the 248 cases reported in the literature, the full-term delivery rate was 9%, the incidence of gestational trophoblastic tumors was 35%, and approximately 21% were associated with assisted reproductive technology.
Salmeri et al. (Meta-analysis) (5)	2025	417	Ultrasound detection rate: 76.0%; early pregnancy detection rate: 52.7%; incidence of symptoms: 80.5%; live birth rate: 46.5%.
An Overview of the PMC System (21)	2025	118 cases at ≥23 weeks	Complete molar pregnancies accounted for 87% of cases, with a median gestational age at delivery of 31.6 weeks and a cesarean section rate of 79%.

The genetic basis of CHM is “fertilization of an empty oocyte,” in which the oocyte nucleus is absent or inactive, and the sperm provides the entire chromosomal complement; the karyotype is typically 46,XX (10, 11). In CHMCF, one conceptus is a normal diploid fetus (46 chromosomes, with 23 chromosomes from each parent), while the other is a complete hydatidiform mole formed when an empty oocyte is fertilized by sperm (22).

In recent years, with the increasing use of assisted reproductive technologies (ART), such as ovulation induction, the incidence of complete hydatidiform mole coexisting with a fetus in a twin gestation has increased. Reports of this condition among ART patients have also increased, and the association between the two has become an important research focus in recent years. Zilberman Sharon et al. (23) performed a systematic review of the literature from 1998 to 2018 and found that, among 120 cases of CHMCF where the conception method was reported, 25 cases (21%) followed ART. A systematic review published in 2024 conducted a systematic search of the PubMed database and identified a total of 26 reported cases of hydatidiform mole with a coexisting live fetus after conception via assisted reproductive technology (ART) (24). Although case numbers remain small, given the rarity of CHMCF, the accumulation of ART-related cases is sufficient to warrant clinical awareness. Additionally, some studies have suggested that the incidence of molar pregnancy was approximately 1/4,300 in fresh cycles, while the estimated incidence in frozen-thawed cycles ranged from 1/2,300 to 1/2,900, suggesting a possible 50%–100% increased risk. This phenomenon may reflect epigenetic changes in embryos during the freezing and thawing process or alterations in endometrial receptivity; however, the underlying mechanism remains uncertain (6). During ART procedures, particularly intracytoplasmic sperm injection (ICSI), superovulation may lead to the formation of enucleated eggs. In addition, meiotic spindle disruption during oocyte manipulation, loss of maternal chromosomal material, and oocyte fragmentation or degeneration may contribute to the occurrence of fertilization of enucleated eggs (22). Procedures including *in vitro* culture and cryopreservation during ART may alter imprinted-gene expression in embryos. Since the formation of a complete hydatidiform mole involves the loss of maternally imprinted gene expression, any factor affecting the stability of genomic imprinting may increase the risk of its development. Although controversy remains regarding the potential malignant outcomes in CHMCF after ART, a case of CHMCF after ICSI reported by Alsaffar et al. (25) in 2025 described a patient who continued gestation under intensive surveillance and delivered a healthy liveborn infant via elective cesarean section at 37 weeks of gestation. This suggests that expectant management of CHMCF after ART is feasible when based on full informed consent and multidisciplinary collaboration. As noted above, some studies indicate that the incidence of hydatidiform mole may be increased during frozen-thawed cycles. This suggests that, although CHMCF is rare, patients should be counselled regarding the possibility of this complication during pre-ART counseling, particularly when multiple embryos are transferred.

Vaginal bleeding is the most common presenting symptom of CHMCF, with a reported frequency of 80.5% (26); bleeding may present as intermittent spotting or as profuse bleeding. Severe hyperemesis gravidarum is another common manifestation. A study published in Guangdong Medicine in 2017 (27) showed that among 12 patients with multiple gestations complicated by hydatidiform mole, 4 developed severe hyperemesis, 10 had severe vaginal bleeding, and 2 reported abdominal pain. The occurrence of hyperemesis gravidarum is closely related to markedly elevated levels of β-hCG stimulating central emetic pathways. Uterine enlargement greater than expected for gestational age is a key clinical sign of CHMCF (28), caused by rapid proliferation and expansion of hydatidiform mole tissue. Serum β-hCG concentrations in patients with CHMCF are typically markedly elevated, often exceeding 200,000 mIU/mL. Extremely high serum β-hCG levels are not only a key diagnostic clue but are also associated with the risk of subsequent gestational trophoblastic neoplasia (GTN) (26). However, clinicians should note that the “hook effect” can lead to false-negative results. When β-hCG concentrations are excessively high, standard immunoassays may become saturated, resulting in falsely low results. In clinical practice, when typical clinical features are present but β-hCG laboratory results are discordant, repeat testing after sample dilution should be considered (29, 30).

Early recognition and accurate diagnosis of CHMCF are essential for optimizing maternal and fetal outcomes. With advances in ultrasonographic technology and greater clinical awareness, the timing of CHMCF diagnosis has been substantially advanced; however, its clinical manifestations remain heterogeneous and nonspecific, creating challenges for early diagnosis. In early gestation, CHMCF often manifests with nonspecific ultrasonography findings and may be misdiagnosed as early intrauterine pregnancy complicated by intrauterine hematoma or fetal demise in one of a twin gestation. An analysis of 9 cases of CHMCF from Peking University Third Hospital (31) showed that 8 patients underwent ultrasonography during early gestation (weeks 6–10). Among these, 5 cases (62.5%) exhibited abnormal sonographic features, characterized by a normal gestational sac, embryo, and fetal heartbeat within the uterine cavity, along with a heterogeneous, irregular, mass-like lesion with intermediate or low echogenicity adjacent to the gestational sac. The key feature at this stage is the presence of an “aberrant echogenic area adjacent to a normal gestational sac”; however, this area lacks the typical honeycomb-like structure and can be misinterpreted as intrauterine hemorrhage or degenerated embryonic tissue. Therefore, patients in whom an aberrant mass is identified within the uterine cavity during early gestation ultrasonography should be advised to undergo a follow-up ultrasonography between 11 and 13 weeks of gestation. The period between 11 and 13 weeks of gestation is the optimal diagnostic window for diagnosing CHMCF. Typical ultrasonography findings include a normally developed fetus and placenta within the uterine cavity, along with the characteristic ultrasonography features of a hydatidiform mole—namely, “honeycomb” or “snowflake” patterns—evident adjacent to the placenta (32). A recent systematic review published by Salmeri et al. in 2025 (including 19 studies with a total of 417 cases of CHMCF) showed that the detection rate in early gestation (before 13 weeks) was 52.7%, and the overall accuracy of ultrasonographic diagnosis was 76.0% (26). Some cases of CHMCF may continue into the second or third trimester or even until term. Liu Liping et al. reported a case of CHMCF resulting from a natural gestation after ovulation induction; honeycomb-like placental changes in the placenta were first identified at 17 weeks of gestation, and the gestation was continued under intensive surveillance until 38 weeks, when a term infant was delivered via cesarean section (14). This case suggests that, for CHMCF diagnosed at a later stage, continuing gestation may be feasible provided that the risks of maternal complications and the status of fetal development are carefully evaluated. However, late diagnosis usually implies longer maternal exposure to hydatidiform mole tissue, which is associated with an increased risk of complications such as preeclampsia, hyperthyroidism, and bleeding. Furthermore, even in cases where ultrasonography findings are strongly suggestive of the condition, some cases still require additional imaging methods to confirm the diagnosis.

Live birth rates for CHMCF vary among studies but appear to have improved over time. A recent meta-analysis published by Salmeri et al. (5) in 2025 (417 cases) reported a live birth rate of 46.5%; Regarding delivery timing, the preterm delivery rate among women with CHMCF is up to 67.8%, the extremely preterm delivery rate (<32 weeks) is 12.4%, and the miscarriage rate is 32.7%. The frequency of maternal complications in CHMCF is markedly higher than in uncomplicated gestations, and the risk of GTN is markedly higher than in singleton complete hydatidiform moles (33). Massardier et al. (34) suggest that complications such as gestational hypertension, vaginal bleeding, and hyperthyroidism during gestation may not significantly affect the risk of post-molar gestational trophoblastic neoplasia (pGTN). Matsui et al. (18) identified severe antepartum bleeding and gestational hypertension as significant risk factors for pGTN. Giorgione et al. (35) found no significant difference in the risk of pGTN between patients who underwent termination and those who continued the gestation (32). A study by Lin et al. (36) similarly found no significant association between the gestational age at termination and the development of pGTN. Therefore, selecting termination of gestation specifically to prevent the development of pGTN appears unjustified.

Serial serum hCG monitoring after molar evacuation is essential for disease surveillance, treatment assessment, and follow-up (37). CHM progresses to GTN in approximately 13%–20% of cases (38). Guidelines recommend weekly/biweekly hCG until three consecutive normal values, then monthly for 6 months after normalization (37). Delayed hCG regression (each additional week to normalization confers a 1.3-fold increased risk), week-2 hCG ≥1400 IU/L, or a week-2/post-evacuation ratio ≥0.02 independently predicts pGTN (39). Multiple studies have shown that heterozygous (dispermic) CHM carries a significantly higher risk of pGTN than homozygous (monospermic) CHM (40, 41). This highlights the importance of STR genotyping with maternal reference DNA to determine zygosity, enabling more precise risk stratification and individualized follow-up in clinical practice. Our patient showed favorable hCG decline, suggesting low malignant risk. However, the lack of maternal blood for STR-based family validation precluded definitive confirmation of the paternal origin of the homozygous alleles, and the patient was lost to follow-up before completing hCG monitoring, leaving both the possibility of long-term pGTN and biparental CHM (BiCHM) unresolved—these represent the main limitations of this report. BiCHM is associated with mutations in NLRP7 or KHDC3L and is an important cause of recurrent hydatidiform mole (42). Given the patient's history of secondary infertility and prior pregnancy, genetic counseling should be considered for future pregnancies to evaluate the recurrence risk. In addition, the original STR electropherograms and the exact number of informative loci could not be ascertained retrospectively, representing a further limitation of the present study.

In summary, CHMCF is rare in clinical practice. Its imaging features are subtle in early gestation, making it difficult to identify and easily confused with hematometra. The period between 11 and 14 weeks of gestation represents the optimal diagnostic window for identifying the characteristic “honeycomb” echo pattern. Management requires multidisciplinary, individualized decision-making based on a comprehensive evaluation of factors such as the mode of conception, maternal age, the risk of complications, including preeclampsia, hyperthyroidism, and bleeding, and the probability of fetal survival. Regardless of whether the gestation is terminated, post-evacuation serum *β*-hCG levels must be closely monitored to ensure that persistent gestational trophoblastic disease is identified and managed promptly.

## Data Availability

The original contributions presented in the study are included in the article/Supplementary Material, further inquiries can be directed to the corresponding authors.
